# Validation of Digital Cytology for Primary Diagnosis Across a Range of Specimen Types

**DOI:** 10.1111/cyt.70063

**Published:** 2026-03-10

**Authors:** Talisa Mistry, Harriet Hunter, Dahmane Oukrif, Sabine Pomplun, Reena Khiroya, Mary Falzon, Tanya Alan, Manuel Rodriguez‐Justo, Adam P. Levine

**Affiliations:** ^1^ Research Department of Pathology UCL Cancer Institute, University College London London UK; ^2^ Department of Cellular Pathology University College London Hospitals NHS Foundation Trust London UK

**Keywords:** cytology, digital, scanner, validation, whole‐slide image

## Abstract

**Objective:**

This study evaluated the diagnostic performance of high‐resolution whole slide imaging (WSI) for primary cytological diagnosis across a broad range of specimen types and preparations.

**Methods:**

In a single‐centre, retrospective validation study, 88 archived cytology cases representative of routine clinical practice were scanned at 40× equivalent magnification (0.23 μm/pixel) using the Hamamatsu NanoZoomer S360MD Slide scanner system. Specimens included gynaecological and non‐gynaecological exfoliative and fine needle aspirate samples, prepared as smears, ThinPreps, cytospins or cell blocks. WSI were each reviewed independently by two expert cytopathologists with minimal prior digital cytology reporting experience, blinded to original light microscopy (LM) diagnoses. Discordant cases underwent LM review. Diagnostic concordance and agreement were assessed using percentage concordance and Cohen's κ (unweighted and weighted, for non‐gynaecological cases only). A post‐study questionnaire captured qualitative feedback.

**Results:**

Of the 88 cases, 65 showed concordance between digital and LM diagnoses. Amongst the remaining 23 cases, 15 demonstrated diagnostic discrepancy by LM. Excluding these, the digital‐LM concordance rate was 95.1%. When all cases were included, concordance ranged from 89.5% for within‐observer analysis to 89.8% when compared with a majority LM diagnosis. Agreement analysis demonstrated substantial to near‐perfect digital‐LM agreement (unweighted κ = 0.86; weighted κ = 0.83–0.95), improving further following exclusion of diagnostically discrepant cases (κ = 0.89–0.97). Inter‐observer agreement was lower than intra‐observer agreement for both digital and LM comparisons. Feedback indicated that image quality was generally good. Challenges included visualising thick smear preparations, three‐dimensional cell clusters, sparse atypical cells and screening gynaecological (cervical) cytology cases. All participants expressed openness to adopting digital cytology.

**Conclusion:**

High‐resolution WSI demonstrated strong diagnostic concordance with traditional LM across a wide range of cytological specimen types and preparations. Despite limited prior experience, digital cytology was considered acceptable and feasible, supporting its integration into routine clinical practice, with appropriate training and quality assurance.

## Graphical abstract

This single‐centre validation study demonstrated strong diagnostic concordance between high‐resolution whole‐slide imaging and traditional light microscopy across diverse cytology specimen types. Even with limited prior experience, pathologists found digital cytology feasible and acceptable for diagnostic use. The graphical abstract was created in BioRender (https://BioRender.com/lsq1pts).

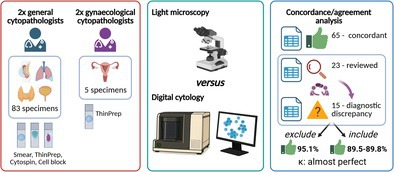

## 1 Introduction

Advancements in digital pathology are redefining the role of conventional light microscopy (LM) in diagnostic practice. The development of high‐throughput, high‐resolution whole‐slide imaging (WSI) systems has enabled large‐scale digitisation of pathology slides, transforming diagnostic workflows [1]. Digital pathology offers several advantages over traditional light microscopy (LM), including efficient image archiving, potential for remote reporting and compatibility with advanced image analysis tools and artificial intelligence (AI) [2, 3]. Several studies have demonstrated high levels of diagnostic concordance between digital WSI and conventional LM for primary and secondary diagnoses in histopathology [4, 5, 6, 7]. These findings have supported regulatory approvals and have helped accelerate the integration of digital pathology into routine clinical practice [8].

Despite momentum in histopathology, the adoption of digital WSI in cytopathology remains relatively limited. Cytology plays a vital role in diagnostic pathways by offering a method for assessment of exfoliative fluid and fine needle aspirate (FNA) samples obtained through minimally invasive approaches from a wide range of specimens and pathologies [9]. Cytological specimens differ from histological sections in both their preparation and interpretation. They are characterised by variable cellularity and often contain three‐dimensional clusters [10]. Interobserver concordance and diagnostic accuracy in cytology are known to be sensitive to preparation‐related factors such as smear thickness, specimen adequacy, fixation artefact and staining inconsistencies. These variables can complicate digital standardisation and image quality [11]. The College of American Pathologists (CAP) has emphasised that the adoption of digital cytology into routine clinical practice depends upon the availability of scanners capable of matching the diagnostic performance of LM [12]. However, thus far, limited validation studies have been undertaken assessing the appropriateness and diagnostic performance of digital WSI platforms in cytopathology for primary diagnosis, particularly across diverse specimen types and preparation methods.

This single‐centre, retrospective validation study aimed to assess the diagnostic performance of the Hamamatsu NanoZoomer S360MD Slide scanner system in a simulated digital cytology workflow across a range of cytological specimen types, preparations and diagnoses. Specifically, this study examines whether diagnoses rendered by expert cytopathologists from the examination of WSI produced by this platform are sufficiently concordant with traditional LM diagnoses to support implementation of digital cytopathology into routine clinical practice.

## 2 Materials and Methods

### 2.1 Study Design

This was a single‐centre retrospective diagnostic validation study aimed at evaluating the diagnostic performance of the Hamamatsu S360MD Slide scanner system for primary cytological diagnosis. Eighty‐eight cases were purposively selected across a range of specimen types, preparations and diagnoses that were representative of common daily practice. The cases included both gynaecological and non‐gynaecological fluid specimens prepared as direct smears, cytospin and ThinPrep preparations. The majority of the cases (86%) were from 2023 and 2024, with the remaining being older (2011–2019). Case selection was undertaken based on the original glass slide diagnosis to reflect common findings for each specimen type (including negative diagnoses). The glass slides of the selected cases were not reviewed prior to inclusion in the study. All cases were archival/historical in nature from at least 6 months prior to the time in which the study was undertaken.

### 2.2 Objectives

The primary objective of this study was to determine the level of diagnostic concordance between high‐resolution WSI and traditional LM across a variety of cytological specimen types, preparations and diagnoses. The secondary objectives were to assess the quality of WSI and to qualitatively determine cytopathologists' attitudes and experiences of digital cytology reporting.

### 2.3 Ethics Statement

Ethical approval for this study was provided by the UCL/H Biobank for Studying Health and Disease (study EC30.22) under NHS HRA delegated authority (REC reference: 20/YH/0088, IRAS ID: 272816). As all patient data were de‐identified, individual patient consent was not required.

### 2.4 Slide Scanning

All slides from each included case were retrieved from the diagnostic archive. Any pen markings were cleaned from the slides prior to scanning. Slide scanning was performed using a Hamamatsu NanoZoomer S360MD Slide scanner system (C13220‐21MDEU) (Hamamatsu, Japan) generating ndpi WSI files. Scanning was performed at 40× equivalent magnification (0.23 μm/pixel) with NZAcquireMD 1.3.0 software. The semi‐automatic scanning function was utilised in which the whole‐slide macro image of the slide is first acquired and the scan area and focus points are automatically positioned and presented for a visual inspection. If needed, these can be manually adjusted prior to high‐resolution scanning. A manual quality review of each generated WSI was performed by the scanning technician. If areas were noted to be out of focus or missed, the slide was re‐scanned with additional focus points added, if required, and manual verification of focus at each point undertaken. The re‐scan status of each slide was not documented; however, it was estimated to be 5%–10%. It was noted that re‐scans were more commonly required for smear preparations and for older slides. Of note, the reporting cytopathologists were not given the opportunity to request re‐scans if they deemed a slide to be poorly scanned which would likely happen in clinical practice. Although the scanner utilised has the capability for z‐stacking (in which multiple images are captured at different focal planes along the z‐axis), this was not performed. The same scanning procedure was utilised for all slides regardless of sample/preparation type. All WSI were anonymised using Hamamatsu's NZMask (U17415‐01 version 1.0.0) WSI anonymisation software. A unique case identifier was assigned to each case to ensure traceability.

### 2.5 Digital Reporting

Digitised cases were each reviewed independently by two UK board (Royal College of Pathology) certified cytopathologists, each with substantial experience (8–35 years) in routine cytology by LM. The reporting cytopathologists had minimal prior experience with digital cytology reporting. Two different cytopathologists reported the gynaecological (cervical) cytology cases (in the United Kingdom, reporting of such cases is separately regulated). WSI were examined on either a 31.5‐in. 8MP (3840 × 2160) or a 16‐in. 4MP (2560 × 1600) monitor using NZViewMD software (version 1.0.4.2). The monitors did not undergo specific calibration and reporting cytopathologists were not given the option to adjust the monitor settings. The monitor type was not recorded prospectively per case, but the reporting cytopathologists described no appreciable difference with the monitor used. The reporting cytopathologists were provided with the same clinical information as was available at the time of the original diagnostic reporting by LM. This included the patients' sex, age, specimen type and clinical history. The latter was of variable detail from none to clinically/radiologically suspected diagnoses. Cytopathologists were blinded to the original LM diagnosis and whether any additional work (e.g., special stains, cell blocks and immunohistochemistry) was performed.

### 2.6 Concordance Analysis

Cases in which there was discordance between the original LM diagnosis and the digital diagnosis for either or both reporting cytopathologist(s) were reviewed by each cytopathologist by LM. A consensus meeting was held to discuss and examine the discrepant cases. Cases showing diagnostic discrepancy by LM were defined as those in which there was no consensus between the diagnoses of the LM reviews and the original LM diagnosis. This classification was rendered for any discrepancies with a potential impact on clinical management and identifies cases with intrinsic cytological diagnostic ambiguity.

**FIGURE 1:**
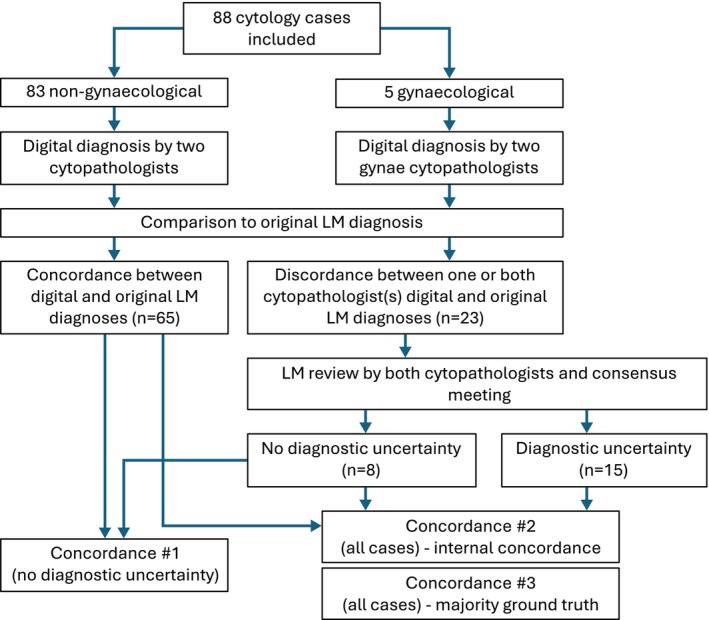
Flow of cases through the study for the concordance analysis. Eighty‐eight cytology cases (83 non‐gynaecological, 5 gynaecological) were included and each underwent digital review by two cytopathologists. Cases were classified according to concordance or discordance to the original light microscopy (LM) diagnosis. Discrepant cases underwent LM review and discussion at a consensus meeting. Three concordance measures were calculated, with or without inclusion of diagnostically uncertain cases.

Three concordance calculations were undertaken. The primary concordance calculation was undertaken excluding cases showing diagnostic discrepancy by LM on the basis that the diagnostic uncertainty could confound the digital‐LM comparison. For the secondary concordance calculations, these cases were included. Internal concordance (digital diagnosis versus review LM diagnosis) for each cytopathologist individually was computed. In addition, a comparison was undertaken between the digital diagnosis and the majority diagnosis from the three LM examinations (the two reviews and the original diagnosis). Cases in which there was no agreement between at least two of the three LM reviews were excluded from the latter analysis. In cases in which there was no diagnostic discrepancy by LM, cytopathologists reviewed the case digitally and by LM to determine the possible reason for the discrepancy (e.g., true inferiority of the digital image or diagnostic error). 95% confidence intervals on percentage concordances were calculated using the Wilson score method for binomial proportions (prop.test function in R (version 4.3.2)) [13]. The flow of cases through the study is shown in Figure 1.

### 2.7 Agreement Analysis

For agreement analysis, each cytological diagnosis was first recoded into one of seven diagnostic categories (defer, inadequate, negative/inflammatory, benign, atypia, suspicious or malignant). Agreement was assessed using Cohen's kappa (κ) with 95% confidence intervals, calculated by both unweighted and weighted analyses. Unweighted κ assessed exact categorical agreement, whilst weighted κ accounted for the ordinal nature of cytological diagnostic categories (benign < atypia < suspicious < malignant), penalising larger diagnostic discrepancies more heavily than adjacent category disagreements. For the latter, benign, negative and inflammatory diagnoses were collapsed into a single benign category to improve stability and reflect similar clinical implications. For a case with granulomatous inflammation in a subset of the diagnoses, subcategories of granulomatous and non‐granulomatous inflammation were utilised for the unweighted analysis, but they were grouped together into the benign category for the weighted analysis.

Agreement analyses were performed on a pairwise basis. Cases were excluded only from specific κ calculations when one (or both) of the two compared ratings was classified as deferred or inadequate. Deferred diagnoses were excluded from all κ analyses; inadequate diagnoses were included in unweighted κ analyses but excluded from weighted analyses. Sensitivity analyses were performed excluding cases flagged as showing diagnostic discrepancy by LM. As gynaecological cytology cases (*n* = 5) were reported by two cytopathologists different from the two cytopathologists reporting the remaining majority of the cases (*n* = 83) and a pairwise individual analysis was utilised, these cases were excluded. Interobserver agreement was assessed separately comparing each cytopathologist's digital diagnosis to each other (WSI‐WSI) and to the LM diagnosis (WSI‐LM). Confidence intervals were estimated using bootstrap resampling (2000 iterations). All statistical analyses were performed using R (version 4.3.2) [13].

### 2.8 Post‐Reporting Questionnaire

Reporting cytopathologists qualitatively assessed image quality and completed a post‐reporting questionnaire to evaluate their experiences with digital cytology reporting. The questionnaire assessed users' prior experience, diagnostic confidence, assessment of image quality, platform usability, technical limitations and future preferences.

## 3 Results

A total of 88 cases were included in the study. The specimen types, preparations and sample numbers are outlined in Table 1. At least four cases were included per specimen type. Overall, there were slightly more smear preparations (*n* = 32, 36.4%) than ThinPrep (*n* = 27, 30.7%) or cytospin (*n* = 25, 28.4%) preparations. The remaining four (4.5%) cases were cell blocks. There were a total of 201 slides across all included cases. This included 112 slides stained with May‐Grünwald Giemsa (MGG) and 59 slides stained with Papanicolaou (Pap). The remaining 30 slides were from the cell blocks and included haematoxylin and eosin‐stained (H&E) slides and immunohistochemically stained slides. On average, each case had a mean of 2.3 and median of 2 slides (range 1–13). 24 cases had at least one MGG and at least one Pap‐stained slide. 29 cases only had one or more Pap slides (mean 1.4) with no MGG slides and 31 cases had one or more MGG slides (mean 2.8) with no Pap slides. The mean age of the patients was 56.3 years and there were slightly more females than males (54.5% female, 45.5% male). The full list of cases included in the study along with their category, specimen type, preparation, number of slides and original LM diagnosis are provided in Supplementary Table 1.

**TABLE 1:** Distribution of specimen types included in the study along with the category, preparation and number. The preparation indicated is the most commonly utilised per specimen type. Where two preparations are given for a specific specimen type, the first was more commonly used except for sputum which was equal between the two.

Category	Specimen	Preparation	Total
Gynaecology	Cervix brush	ThinPrep	5
Respiratory	Sputum	ThinPrep/Smear	6
	Salivary gland FNA	Smear	5
	Bronchial brush/wash	ThinPrep/Smear	5
Body cavity	Pleural fluid	Cytospin	7
	Ascitic fluid	Cytospin	6
	Pleural/ascitic fluid	Cell block	4
Central nervous system	Cerebrospinal fluid	Cytospin	6
Joint	Synovial fluid	Cytospin	4
Urinary	Urine	ThinPrep	7
Mammary	Nipple discharge	Smear	5
	Breast FNA	Smear/Cytospin	7
Thyroid	Thyroid FNA	Smear	7
Hepatobiliary	Common bile duct brush	ThinPrep	5
	Pancreas FNA	ThinPrep/Cytospin	4
Haematopathology	Lymph node FNA	Smear	5
**Total**			**88**

For each of the 88 cases examined, concordance comparisons for the two reviewing cytopathologists per case were considered independently. Across the 88 cases, 65 showed concordance between the two reviewing cytopathologists' digital diagnoses and the original LM diagnosis. In 23 cases, either one or both of the reviewing cytopathologists' digital diagnoses were discordant with the original LM diagnosis, necessitating a LM review. In eight of the 23 reviewed cases, there was diagnostic concordance by LM. However, in the remaining 15, either one or both of the reviewing cytopathologists were discordant with each other and/or the original LM diagnosis. These cases were classified as showing diagnostic discrepancy by LM and assumed to be less reliable and informative for digital‐LM comparisons. The grade of the diagnostic discrepancies observed between the original LM and review diagnoses based on clinical significance was considered to be major (crossing malignant—atypia/benign) in six and minor in nine.

Examining the digital diagnoses for the eight discordant cases not showing diagnostic discrepancy by LM, one cytopathologist made three deferrals, had four discordant comparisons (three clinically significant) and one concordant comparison. The other cytopathologist had three discordant comparisons (all clinically significant) and five concordant comparisons (Table 2). The total number of discordant comparisons was therefore seven out of 143 comparisons (two comparisons for each of 73 cases, 88 cases minus 15 showing diagnostic discrepancy by LM, with three deferrals excluded). The concordance rate amongst the cases not showing diagnostic discrepancy by LM was thus 95.1% (136/143, 95% CI: 89.8%–97.8%) (Table 3). On consensus meeting review of these seven discordant comparisons, the cytopathologists agreed that in all cases the diagnostic features for the LM diagnosis were apparent on the WSI and that the cause of the discordance was interpretative (diagnostic error) and not due to inferior image quality (examples are shown in Figure S1).

**TABLE 2:** Digital diagnoses as compared with light microscopy (LM) diagnosis for the eight cases not showing diagnostic discrepancy by LM (i.e., with consensus amongst the LM reviews and the original LM diagnosis).

Specimen	LM diagnosis	Digital diagnosis	
		Cytopathologist 1	Cytopathologist 2
Cerebrospinal fluid	Metastatic adenocarcinoma	Insufficient material*	Concordant
Common bile duct brushing	Negative for malignancy	Concordant	Atypical cells present*
Lymph node FNA	Metastatic squamous cell carcinoma	Too thick‐defer	Concordant
Lymph node FNA	Non‐necrotising granulomatous inflammation	Reactive*	Concordant
Pleural fluid	Lymphocytic effusion	Small cell carcinoma*	Concordant
Sputum	Squamous cell carcinoma	Too thick‐defer	Concordant
Sputum	Squamous cell carcinoma	Too thick‐defer	Negative for malignancy*
Bronchial brushing	Adenocarcinoma	Negative for malignancy*	Negative for malignancy*

Abbreviation: FNA: Fine needle aspirate.

* Discordances.

**TABLE 3:** Concordance rate between digital and light microscopy (LM) diagnoses calculated using the three different methods. In the first, only cases not showing diagnostic discrepancy by LM are considered. In the second and third, cases showing diagnostic discrepancy by LM are included. In the second, internal concordance is considered (digital‐LM for each cytopathologist) whereas in the third, the concordance was calculated between the digital diagnosis and a majority ground truth LM diagnosis. Comparisons were considered for the two reviewing cytopathologists per case independently. 95% CI: 95% confidence intervals.

Calculation	Diagnostic discrepancy by LM	Method	Total comparisons	Deferrals	Concordant	Concordance rate (95% CI)
1	Excluded	Overall	143	3	136	95.1% (89.8%–97.8%)
2	Included	Internal	172	4	154	89.5% (83.7%–93.5%)
3	Included	Majority ground truth	166	4	149	89.8% (83.9%–93.7%)

In addition, the data were analysed including the 15 cases showing diagnostic discrepancy by LM. When comparing each cytopathologists' digital diagnosis to their own LM diagnosis (or the original LM diagnosis for non‐discordant cases) there were 18 discordant comparisons and 24 concordant comparisons (a total of 154 concordant comparisons including the 65 cases not showing diagnostic discrepancy by LM, each examined twice). There was one additional deferral (plus the three previously described) such that there were 172 comparisons in total. The overall concordance rate was 89.5% (154/172, 95% CI: 83.7%–93.5%). Of the additional 11 discordances (beyond the seven when examining only cases not showing diagnostic discrepancy by LM), ten were deemed clinically significant and one was not.

The concordance was also analysed comparing the digital diagnoses to a majority LM diagnosis (as opposed to the within‐cytopathologist assessment). In three cases, each of the three LM diagnoses (comprising the original LM diagnosis and the two LM study reviews) yielded a differing diagnosis such that no majority diagnosis could be declared. Following the exclusion of these and four deferrals, there were a total of 166 comparisons. Amongst these, there were 17 discordances yielding a concordance rate of 89.8% (149/166, 95% CI: 83.9%–93.7%). A full breakdown of the diagnoses and concordance assessments for the 23 discordant cases with and without diagnostic discrepancy by LM is provided in Supplementary Table 2.

The gynaecological (cervical) cytology cases were included in the calculations but, as they are considered a distinct specimen type with a different reporting process, have also been described separately. Of the five cases, two showed perfect concordance between the reporting cytopathologists and the original LM diagnoses. The remaining three were reviewed by LM and showed diagnostic discrepancy (Table 4). However, of the six comparisons for these three cases, all but two showed within‐cytopathologist concordance, with the discordance cases being between negative and borderline dyskaryosis. One case was originally reported by LM as showing high‐grade dyskaryosis, but on review by both digital and LM was either classified as borderline dyskaryosis or negative. This case had numerous air bubbles underneath the coverslip, likely obscuring the previously identified atypical cells.

**TABLE 4:** Digital diagnoses as compared with light microscopy (LM) diagnosis for three gynaecological (cervical) cytology cases showing diagnostic discrepancy by LM (lack of consensus amongst the LM reviews and the original LM diagnosis).

Original LM diagnosis	Cytopathologist 1		Cytopathologist 2	
	Digital diagnosis	LM diagnosis	Digital diagnosis	LM diagnosis
Low‐grade dyskaryosis	Negative	Borderline dyskaryosis	Low‐grade dyskaryosis	Low‐grade dyskaryosis
High‐grade dyskaryosis	Moderate dyskaryosis	Moderate dyskaryosis	Moderate dyskaryosis	Moderate dyskaryosis
High‐grade dyskaryosis*	Borderline dyskaryosis	Borderline dyskaryosis	Borderline dyskaryosis	Negative

* Numerous air bubbles likely obscuring the previously identified atypical cells.

In addition to percentage concordance, diagnostic agreement was assessed using Cohen's κ calculated on recoded categorical diagnoses for non‐gynaecological cases (Table 5). Comparisons were undertaken between each cytopathologist's digital and LM diagnoses and between the two cytopathologists' digital and LM diagnoses. For digital–LM comparisons, the reviewed LM diagnosis was used where available (23 cases), and the original LM diagnosis was used for the remaining cases on a per‐cytopathologist basis.

**TABLE 5:** Cohen's kappa agreement between digital whole‐slide image (WSI) diagnoses and original light microscopy (LM) diagnoses (using recoded categories) for two cytopathologists (C1 and C2) examining 83 non‐gynaecological cytology cases. Comparisons are shown with and without cases showing diagnostic discrepancy by LM excluded. Unweighted and weighted analyses have been performed, the latter penalising larger diagnostic discrepancies more heavily than adjacent category disagreements. Deferrals and inadequate cases were excluded.

Weighting	Diagnostic discrepancy by LM	Cohen's kappa (95% CI)			
		C1 digital–LM	C2 digital–LM	C1 LM–C2 LM	C1 digital–C2 digital
Unweighted	Included	0.86 (0.76–0.95)	0.86 (0.76–0.95)	0.73 (0.61–0.83)	0.72 (0.60–0.83)
	Excluded	0.89 (0.79–0.98)	0.94 (0.86–1.00)	0.90 (0.81–0.98)	0.87 (0.77–0.96)
Weighted	Included	0.95 (0.89–0.99)	0.83 (0.65–0.95)	0.82 (0.68–0.92)	0.71 (0.51–0.87)
	Excluded	0.97 (0.91–1.00)	0.91 (0.75–1.00)	0.94 (0.88–0.99)	0.93 (0.85–0.98)

Intra‐observer agreement between digital and LM diagnoses was high for both cytopathologists, with unweighted κ values of 0.86 and weighted κ values ranging from 0.83 to 0.95. Exclusion of cases showing diagnostic discrepancy by LM resulted in further improvement in intra‐observer agreement (κ 0.89–0.97). Inter‐observer agreement was generally lower than intra‐observer agreement for both digital (WSI–WSI) and LM–LM comparisons when all cases were included (WSI–WSI: unweighted κ 0.72, weighted κ 0.71; LM–LM: unweighted κ 0.73, weighted κ 0.82). However, inter‐observer κ values increased substantially following exclusion of cases showing diagnostic discrepancy by LM (WSI–WSI: unweighted κ 0.87, weighted κ 0.93; LM–LM: unweighted κ 0.90, weighted κ 0.94), approaching intra‐observer levels.

Overall, agreement between digital and LM diagnoses was comparable to, and in some analyses exceeded, inter‐observer agreement within each modality, indicating that observed digital–LM discrepancies primarily reflect interpretive variability rather than modality‐specific effects. An important caveat is that full per‐cytopathologist diagnostic data were available for digital interpretation for all cases, whereas per‐cytopathologist LM interpretations were available only for the subset of cases that underwent LM review, with the original LM diagnosis used for the remaining cases. This potentially inflates κ due to structural concordance.

Cases showing diagnostic discrepancy by LM and/or discordance occurred in all specimen types and preparations except for cell blocks and synovial fluid (Supplementary Table 2). A greater frequency of cases showing diagnostic discrepancy by LM and/or discordance (at least two cases) was seen in nipple discharge, cervical brush, lymph node FNA, common bile duct brushing, breast FNA, urine and sputum. In addition, the majority (3 of the 4) cases in which the cytopathologist deferred to glass were smear preparations. Some but not all of the discordant cases were noted to be paucicellular or with thickly spread smear preparations and three‐dimensional clusters of cells that were difficult to visualise. However, the sample sizes are too small to make a definitive assessment of the relationship between quality/diagnostic ability and specimen type/preparation.

Feedback provided by the four reporting cytopathologists (two who reported the 83 non‐gynaecological cases and two who reported the five gynaecological cases) indicated that all had a generally positive experience with digital cytology. This is despite having had limited prior exposure, with three of the four having never reported cytology cases digitally before. The questionnaire findings have been summarised below in a domain‐specific manner with illustrative free‐text response comments provided in Text S1.

**Image quality and visualisation**. In general, all cytopathologists felt the images were acceptable for diagnostic purposes and largely of good or excellent quality, with only occasional reports of blurry or out‐of‐focus areas. Challenges relating to visualisation included difficulty assessing thickly spread smear preparations, three‐dimensional clusters of cells, and identifying sparse atypical cells.

**Navigation, ergonomics and usability**. The digital viewer was considered easy to use, adequate and responsive. One comment explicitly noted that there was no lag or pixellation. Difficulties were described with navigation in relation to scanning the whole slide digitally. This was particularly an issue for the gynaecological (cervical) cytology cases, which are screening rather than diagnostic in nature, and where regimented examination is required. This was found to be cumbersome and time‐consuming to do digitally. No concerns were raised regarding colour fidelity, although this was not specifically enquired about. Improvement or adaptation over time was not assessed or commented on.

**Diagnostic confidence**. The reporting cytopathologists were moderately confident with their digital diagnoses; however, all reported greater confidence when using traditional glass slides.

**Workflow and turnaround time**. Turnaround time, although not recorded on a per‐case basis, was subjectively perceived to be either unchanged or increased when reporting digitally.

**Suitability for different cytology workflows and future use**. The challenges associated with screening were particularly evident for gynaecological (cervical) cytology cases and digital reporting of these cases was therefore considered less practical in its current format. This would be partly addressed if slides were to be pre‐screened and annotated (as per current practice in the UK cervical screening programme) or first reviewed by AI. There was broad openness to considering the adoption of digital cytology for both primary diagnosis and second opinions, with the above caveats noted.

## 4 Discussion

This study evaluated the diagnostic concordance between WSI and traditional LM across a diverse range of cytological specimen types. Our findings demonstrate approximately 95% concordance between digital and LM in cases not showing diagnostic discrepancy by LM (> 60 cases), meeting the validation requirements of the CAP guidelines for digital pathology. Exclusion of cases showing diagnostic discrepancy by LM was undertaken on the basis that such cases represent intrinsic diagnostic ambiguity a recognised challenge in cytopathology with interobserver variability and diagnostic subjectivity [14] rather than shortcomings of either visualisation modality. Exclusion of cases lacking diagnostic consensus has been implemented by others for digital pathology validation [15]. However, it is important to recognise that exclusion of such cases may inflate apparent concordance and agreement estimates, and results should therefore be interpreted in this context. It is noted that some of these excluded cases may be considered more ‘difficult’ and thus excluding them could overestimate the real‐world digital diagnostic capability. However, some of these cases may simply be subject to greater inter‐observer variation, e.g., cases with low cellularity where different cytopathologists require different thresholds to reach a diagnosis. In real life clinical practice, it is likely that cytopathologists may defer to glass or seek a second opinion from a colleague which is considerably easier to do in a digital workflow. Regardless, even when cases showing diagnostic discrepancy by LM were included, overall concordance remained approximately 90% across two alternative analyses.

In addition to a high percentage concordance, agreement analysis using Cohen's κ demonstrated substantial to near‐perfect agreement between digital and LM diagnostic categories in non‐gynaecological cytology cases (gynaecological cases were excluded as they were reported by different cytopathologists). Intra‐observer agreement between digital and LM diagnoses was high for both cytopathologists. Inter‐observer agreement was generally lower than intra‐observer agreement when all cases were included, both for digital (WSI–WSI) and LM–LM comparisons, consistent with the well‐recognised interobserver variability inherent to cytopathology. Importantly, κ values for both digital and LM inter‐observer comparisons increased substantially following exclusion of cases showing diagnostic discrepancy by LM, approaching intra‐observer levels. These findings support the interpretation that a small subset of intrinsically ambiguous cases disproportionately contributes to apparent discordance rather than reflecting limitations of digital imaging itself. Interpretation of LM–LM agreement warrants additional caution, as per‐cytopathologist LM diagnoses were available only for a subset of cases, with the original LM diagnosis used for the remaining cases, potentially introducing a degree of structured concordance.

The concordance and agreement analyses demonstrating excellent performance are despite the study cytopathologists having had limited experience with digital cytopathology reporting. There is likely an early learning‐curve effect whereby digitally naïve cytopathologists may initially underperform relative to expected steady‐state practice. Such a learning curve may also help explain qualitative feedback indicating increased navigation effort and perceived reporting burden when using digital slides. It is reasonable to assume that with increased familiarity, targeted training and workflow optimisation, both diagnostic confidence and efficiency would improve, and the incidence of interpretive discordance may decrease.

Several previous studies have examined the concordance of digital cytology compared with LM. A 2019 systematic review identified 19 studies with an average concordance of 84.1% and intra‐observer concordance reaching 92.5% [16]. A range of scanners were utilised. Most of the studies included were restricted to a single cytological sample type and/or preparation type, often with a limited sample size (median 22). Since 2019, several additional studies have been published. The majority of these only examined a single specimen type, most commonly cervical cytology [17, 18, 19] but also lymph node FNA [20] and lymph node imprints [21]. A small number of studies have examined multiple specimen types, as per the present study. A 2023 study [22] examined 383 cases across a range of specimen types, scanned with an Aperio GT450 (Leica Biosystems). They achieved 98.7% concordance, with only five cases reported as ‘majorly discordant’. Although this study covered a wide range of specimen types the distribution was uneven with 240 cervical cytology cases, 73 thyroid FNA cases and 70 of all other types (median 4 per category). A more recent 2025 study [23] examined 110 cases across a range of specimen types (median 5 per category) scanned with a Hamamatsu NanoZoomer S210 Slide scanner. The majority (83%) of the specimens were smears with the remaining (17%) being cytospin preparations and the overall concordance rate was 87.3%. Several cytology national quality assurance programmes now include digital cytology [24, 25]. In 2024, the American Society of Cytopathology Digital Cytology Task Force published a white paper summarising the current state of digital cytology along with best practice recommendations for incorporating digital cytology into practice [26].

Seven cases in our study not showing diagnostic discrepancy by LM showed clinically significant digital‐LM discordance (see Table 2). Some of these were false negative results: missing non‐necrotising granulomatous inflammation in a lymph node, adenocarcinoma cells in cerebrospinal fluid and bronchial brushings, and squamous cell carcinoma in a sputum sample. False positive results included the identification of atypical cells in a negative bile duct brushing, and misdiagnosing a lymphocyte rich effusion as small cell carcinoma (interestingly, this patient did subsequently have a lung small cell carcinoma diagnosed by biopsy). However, it is important to note several points. First, as stated, the study cytopathologists had limited experience of digital cytopathology reporting, with three of the four having never reported cytology cases digitally before. In UK clinical practice, RCPath guidance stipulates that pathologists are required to first undergo a strictly monitored period of double reporting with both modalities for training purposes [27]. Second, the design of this study encouraged pathologists to provide a definitive diagnosis where possible, despite the lack of experience and lack of access to real‐time radiological and clinical data, and further diagnostic testing such as immunohistochemistry. As noted above, in real‐life clinical practice, it is likely that further consideration or showing of challenging cases to a colleague would be undertaken prior to authorisation. Diagnostic certainty was not prospectively recorded and thus analyses stratified on this were not possible, although the potential value of this is appreciated. At least one discordant case was affected by poor slide quality due to air bubbles likely introduced after the original LM diagnosis, highlighting the heterogeneity of archival cytology material and the potential impact of pre‐analytical factors on digital review.

Upon re‐review of the discordant cases, the study cytopathologists agreed that the morphological features required for the LM diagnoses were visible in the digital images, and that the observed discrepancies were primarily attributable to interpretative differences rather than limitations in image resolution or scan quality. This interpretation is supported by the agreement analysis, which demonstrated higher intra‐observer agreement between digital and LM diagnoses than inter‐observer agreement within either modality. When all cases were included, inter‐observer agreement was lower for both digital (WSI–WSI) and LM–LM comparisons, consistent with the recognised interobserver variability inherent to cytopathology. However, interpretation of LM agreement is caveated by the fact that per‐cytopathologist LM diagnoses were only available for a subset of cases, with the original LM diagnosis used for the remaining cases, potentially introducing a degree of structured concordance, as stated above. Importantly, κ values for both digital and LM inter‐observer comparisons increased following exclusion of cases showing diagnostic discrepancy by LM, approaching intra‐observer levels. Taken together, these findings suggest that many digital–LM discordances reflect intrinsic diagnostic variability rather than modality‐specific limitations, whilst acknowledging the constraints imposed by the study design.

Another issue encountered was deferral of specimens deemed to be too thick to interpret. Whilst this feature is technically attributable to poor sample preparation rather than the quality of the scanning, when using LM the focus can be adjusted to move up and down through the cells in a three‐dimensional cluster. This is a well‐described challenge with digital cytology. One potential solution is the use of z‐stacking [10] (scanning each sample at multiple depths that can be scrolled through on the digital image viewer as though adjusting the plane of focus on LM). Although many scanners have z‐stacking capability, including the one utilised in this study, there are significant drawbacks such as increased scanning time, multiplication of the file size for each additional layer scanned, and complexities in navigation, especially for users unfamiliar with digital interfaces. Currently there is no consensus on the added value of z‐stacking and its appropriateness for clinical use [26].

We observed that more smear preparations than cytospin or ThinPrep preparations were deferred, although the sample size was insufficient for the assessment of statistical significance. This is an expected finding, since in the preparation process for liquid‐based cytology (LBC) preparations, cells are spread evenly, and large clusters are excluded. It has been suggested [22] that the many of specimens for which smears are currently utilised are just as amenable to liquid‐based techniques. The future expansion of digital cytopathology may lead to an increased use of LBC in preference to direct smears. Furthermore, computational approaches such as semantic or extended focusing may be of value [28]. For now, in the small proportion of cases in which there are three‐dimensional clusters of cells or other features that are difficult to visualise digitally, and diagnostic ability is limited, such cases can be deferred to glass.

This study has several advantages over previous work including the breadth of specimen types and preparations included. To achieve this breadth, the number of samples of any given type was consequently low. It is a major limitation of this work, preventing interpretation of performance for specific sample types. This is relevant as it has been noted that digital‐LM concordance can vary across cytological samples and preparations [22]. In addition, no objective quantifiable data were collected on the effect on reporting times and workflow, although this is likely to change as cytopathologists gain digital experience, so any measurements would have had limited significance. Furthermore, this was a single‐centre study using a single scanner platform, which may limit generalisability to other institutional settings. Potential Hawthorne effects, whereby awareness of participation in a validation study influences reporting behaviour, cannot be excluded.

The findings of this study cautiously support the use of WSI as a viable alternative to LM for the interpretation of a wide range of cytopathology specimens in routine clinical practice, with supporting data from the concordance metric and qualitative feedback from participants. As with, digital histopathology, the digitisation of cytopathology offers significant potential benefits including the ease of sharing of pathology slides across geographical areas (for second opinions, and in response to workforce demands), flexible working practices and teaching [3]. Importantly, digitisation is a necessary step towards the use of AI tools to aid assessment and reporting. This is likely to be particularly useful for screening for sparse atypical cells, e.g., in cervical cytology, where the entire slide must be examined systematically. AI tools for this purpose have already been developed [29, 30, 31] and their introduction would likely result in improvements in efficiency and diagnostic accuracy.

## 5 Conclusion

This study has demonstrated that high‐resolution WSI achieves strong diagnostic concordance with conventional LM across a wide spectrum of cytological specimen types and preparations. Despite limited prior experience amongst reporting cytopathologists, digital diagnosis proved both feasible and acceptable, with most discordances attributable to interpretative rather than technical factors. Agreement analysis further indicates that digital–LM agreement is comparable to inter‐observer agreement within each modality, suggesting that observed discrepancies largely reflect intrinsic diagnostic variability rather than modality‐specific effects. These findings support the integration of digital cytology into routine diagnostic workflows, provided that adequate training and quality assurance measures are implemented. Ongoing optimisation of scanning protocols, specimen preparation and the use of advanced tools such as z‐stacking or computational focusing may further enhance diagnostic reliability. Importantly, the adoption of digital cytology establishes the foundation for the application of AI to assist in screening, triage and pattern recognition, offering significant potential to improve efficiency and diagnostic accuracy in future practice. Further work is required to evaluate training effects, the effect of digital cytology on reporting workflow times and the use of AI‐assisted screening in a prospective setting.

## Author Contributions

A.P.L., S.P., and M.R.‐J., conceived the study. A.P.L., selected the cases. T.M., and D.O., retrieved, processed and scanned the slides. S.P., R.K., M.F. and T.A., reported the cases. A.P.L., analysed the data. T.M., H.H., and A.P.L., drafted the manuscript. All authors reviewed and approved the final version of the manuscript.

## Funding

This work was supported by The Jean Shanks Foundation and The Pathological Society of Great Britain & Ireland, and Hamamatsu Photonics K.K.

## Conflicts of Interest

Hamamatsu provided funding to support the study. They had no role in selecting the cases, the review and diagnosis, analysis of the results or drafting the manuscript. The authors declare no other conflicts of interest.

## Supporting information


**Figure S1:** Example images from cases showing diagnostic discordance.

**Text S1:** Illustrative comments from the post‐reporting questionnaire.

**Supplementary Table 1:** Full list of cases included in the study.

**Supplementary Table 2:** Details for discordant cases.

## Data Availability

Research data are not shared.
