# Supplementary material for: Validation of Digital Cytology for Primary Diagnosis Across a Range of Specimen Types
Source: Cytopathology. 2026 Mar 10;37(3):222–31. doi: 10.1111/cyt.70063 (PMC13059545; doi:10.1111/cyt.70063)
Supplement: Supplementary file 1 — Figure S1: Example images from cases showing diagnostic discordance. [file CYT-37-222-s002.docx]

**Supplementary Figure 1**

| **A**  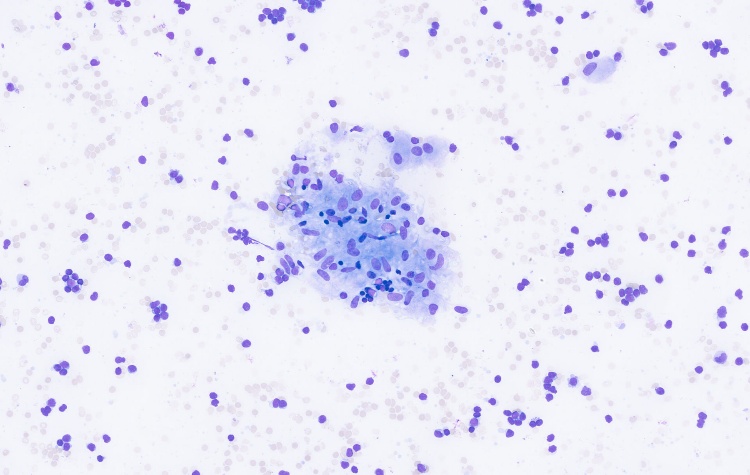 | **B**  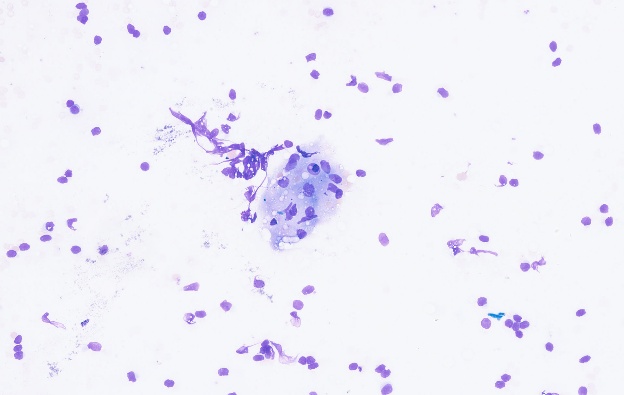 |
| --- | --- |
| **Case #40, lymph node FNA.** Discordance: granulomatous inflammation versus non-granulomatous inflammation. The images show two clusters of epithelioid histocytes, one larger (in A) and one smaller and looser (in B). The group in A is convincingly diagnostic of a granuloma but was only seen infrequently in this case. The smaller and looser group (in B) may or may not be considered sufficient for a diagnosis of granulomatous inflammation by different cytopathologists. | |

| **C**  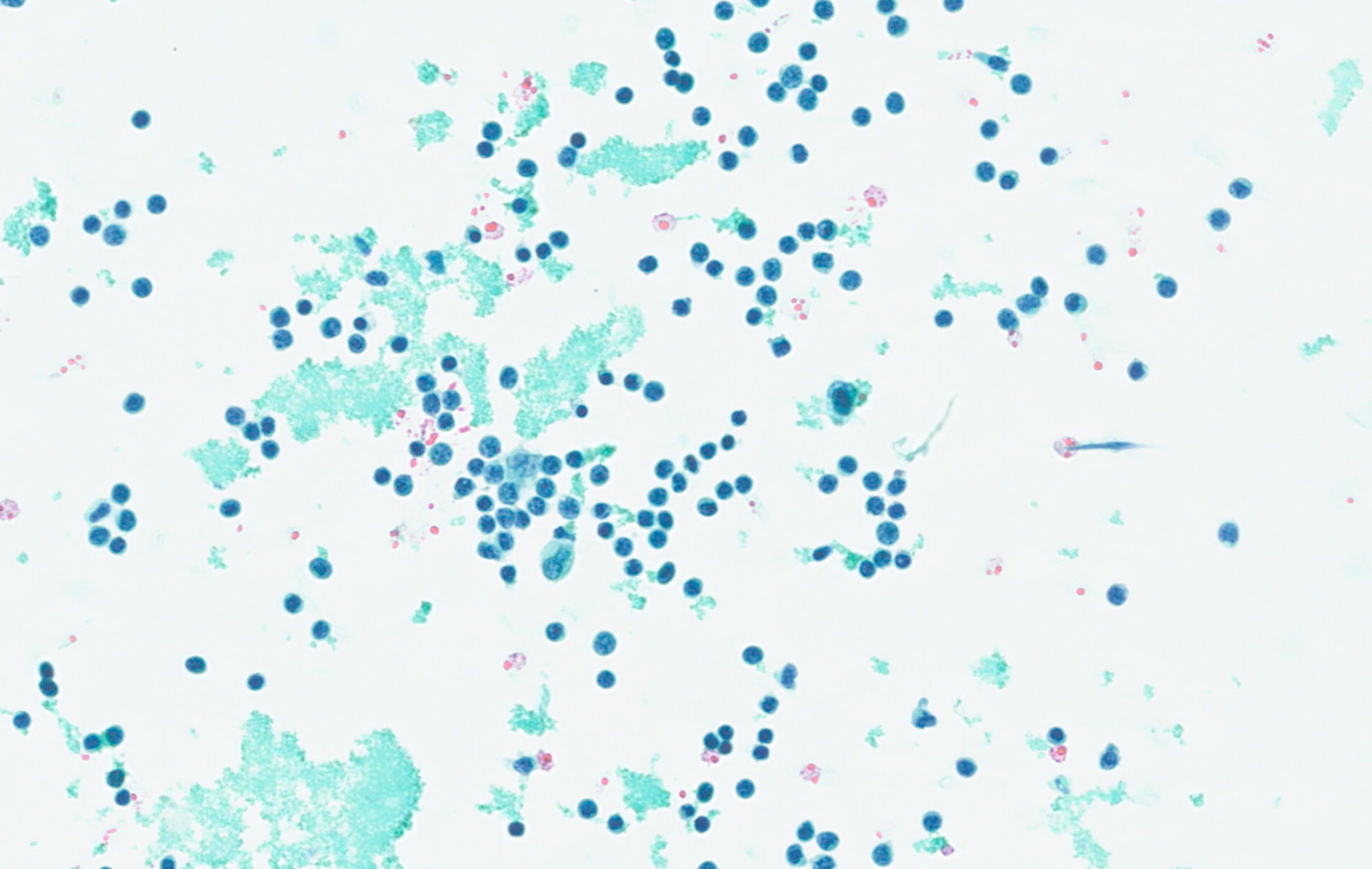 | **D**  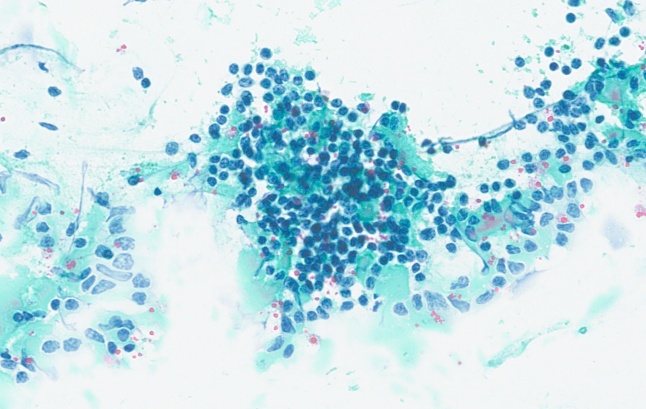 |
| --- | --- |
| **Case #53, pleural fluid.** Discordance: small cell carcinoma versus lymphocytic effusion. In C, there are dispersed medium sized cells with high nuclear cytoplasmic ratio, interpreted by one cytopathologist as lymphocytes. In D, similar cells in a different field show an aggregate/clustering arrangement with a suggestion of nuclear moulding. One cytopathologist favoured small cell carcinoma. Small cell carcinoma versus lymphocytic effusion is a recognised area of diagnostic difficulty in cytology. | |

| **E**  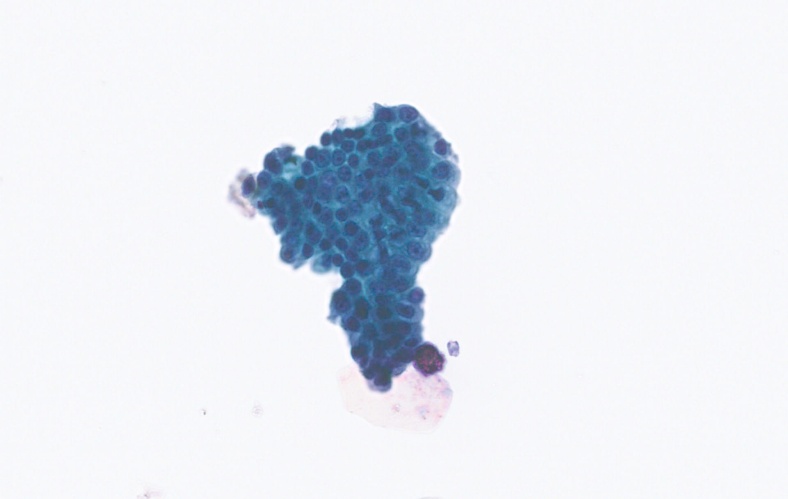 | **F**  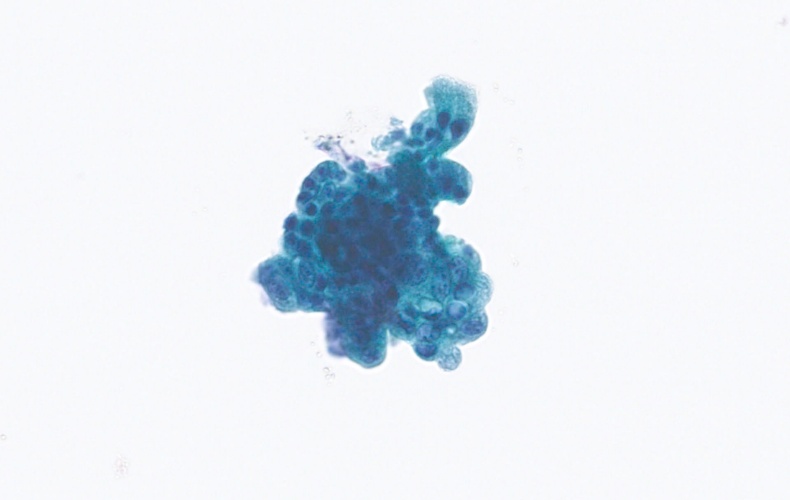 |
| --- | --- |
| **Case #29, common bile duct brushing.** Discordance: No malignant cells seen versus adenocarcinoma. The images show groups of cohesive ductal epithelial cells showing variable architectural and nuclear atypia. Bile duct cytology is an area of high interobserver variation in diagnostic cytopathology with different thresholds for malignancy. | |
