# Supplementary material for: Validation of Digital Cytology for Primary Diagnosis Across a Range of Specimen Types
Source: Cytopathology. 2026 Mar 10;37(3):222–31. doi: 10.1111/cyt.70063 (PMC13059545; doi:10.1111/cyt.70063)
Supplement: Supplementary file 2 — Text S1: Illustrative comments from the post‐reporting questionnaire. [file CYT-37-222-s004.docx]

**Supplementary Text 1**

The following comments are provided to illustrate themes arising from an exploratory, study-specific questionnaire and are not intended to represent exhaustive or quantifiable findings. The questionnaire also included additional items using ordinal response scales; these results are summarised narratively in the main text of the manuscript.

Image quality and focus

- “Really good quality scans”.
- “Clear with no lag or pixellation”.
- “Thicker smears harder to focus”.
- “Thickly spread FNA slides were difficult [to visualise]”.
- “3 dimensional clusters where you need to go up and down [were difficult to visualise]”.

Diagnostic confidence

- “Overall, I feel my glass diagnoses were more accurate”.
- “Nothing is better than [glass] slides for gynae cytology”.

Usability, navigation, and ergonomics

- “Difficult to screen over large areas”.
- “Too many cells on the screen”.
- “Scanning the whole slide is challenging”.
- “It is tempting to go into really high power too quickly”.

Workflow suitability and turnaround time

- “I think it works well and should be adopted”.
- “[For gynae cytology] it is probably unworkable in its current format for screening due to volumes of samples and time taken”.
- “I would love to use digital but not in this format for gynae cytology ... tiles [pre-selected by AI] would be preferrable”.

Suggested improvements / future directions

- “Integration of AI would be beneficial”.
- “Sharper microfocus [would be beneficial]”.
- “FNA sample preparation might need to be changed to accommodate digital scanning”.
